# A novel TGFβ1–Hs3st2–tau axis regulates tau pathology and synaptic integrity

**DOI:** 10.3389/fnins.2025.1726022

**Published:** 2026-02-23

**Authors:** Rafael Castillo-Negrete, Heloise Merrick, Sethupathiraj Selvaraj, Minh Bao Huynh, Xavier Laffray, Ajitha Thuraisamy, Martin Diener, Peter Jedlicka, Mohand Ouidir Ouidja, Dulce Papy-Garcia

**Affiliations:** 1 Glycobiology, Cell Growth and Tissue Repair Research Unit (Gly-CRRET), University Paris Est Creteil (UPEC), Creteil, France; 2 International Giessen Graduate Centre for the Life Sciences (GGL), Justus-Liebig-University Giessen, Giessen, Germany; 3 Translational Neuroscience Network Giessen, Germany; 4 Institute for Veterinary Physiology and Biochemistry, Justus-Liebig-University Giessen, Giessen, Germany; 5 Computer-Based Modelling in the field of 3R Animal Protection, ICAR3R, Faculty of Medicine, Justus-Liebig-University Giessen, Giessen, Germany; 6 Institute of Clinical Neuroanatomy, Neuroscience Center, Goethe University, Frankfurt/Main, Germany

**Keywords:** AD-related tauopathy, Hs3st2, neurodegeneration, synapse, tau, TGFβ1

## Abstract

**Introduction:**

Tauopathies are a group of neurodegenerative diseases characterized by the pathological accumulation of tau protein. The hippocampus, a brain region crucial for learning and memory, is particularly susceptible to tau-induced damage. However, the molecular mechanisms underlying this vulnerability remain poorly understood. Here, we identified a novel TGFβ1–HS3ST2–tau signaling axis involved in tau pathology and synaptic impairment.

**Methods:**

We used primary hippocampal neurons from a transgenic mouse model of tauopathy to investigate the relevance of TGFβ1 signaling on *Hs3st* expression and tau pathology. Loss-of-function (LOF) experiments targeting the neural *Hs3st2* were conducted and cells were analyzed using transcriptomics, immunoblotting, and immunohistochemistry.

**Results:**

TGFβ1 signaling enhanced *Hs3st* gene expression, linking neuroinflammation to altered HS biosynthesis. TGFβ1 was shown to regulate tau hyperphosphorylation and oligomerization through the accumulation of 3-*O*-sulfated heparan sulfate (3S-HS) made by *Hs3st2*. *Hs3st2* LOF significantly reduced 3S-HS levels, tau pathology, and synaptic alterations in hippocampal neurons.

**Discussion:**

These findings define a new TGFβ1–*Hs3st2*–tau axis in the hippocampus and highlight 3S-HS as a key modulator of tau pathology and synaptic dysfunction. Targeting this pathway may offer new therapeutic opportunities in tauopathies and related neurodegenerative disorders.

## Introduction

During neurodegeneration, the progressive loss of neuronal structure or function is driven by the deposition of abnormal proteins like the microtubule-associated protein tau. This protein plays a central role in the development of tauopathies such as Alzheimer’s disease (AD) (Ballatore et al., 2007; Blair et al., 2013; Wang and Mandelkow, 2016). In neurodegenerative diseases, tau abnormal phosphorylation and aggregation contributes to tau deposition and synaptic loss which leads to neuronal dysfunction and memory impairment (Cummings, 2004; Robbins et al., 2021). Although tau pathology correlates strongly with cognitive decline, particularly due to its disruptive effects on synaptic integrity and neuronal communication (Arriagada et al., 1992; Iqbal et al., 2005), other molecular players involved in these processes remain poorly known. Heparan sulfate proteoglycans (HSPG) are glycosylated core proteins bearing heparan sulfates (HS) chains, which play a critical role in regulating synaptic signaling, trafficking, stability and plasticity (Zhang P. et al., 2018). They can modulate the interactions of growth factors and cytokines with their receptors and extracellular matrix (ECM) components (Van Horssen et al., 2003). HS are typically located on the cell surface and in the ECM (Li and Kusche-Gullberg, 2016). Interestingly, in AD they also colocalize intracellularly with tau in intracellular neurofibrillary tangles (NFTs) (Goedert et al., 1996; Hasegawa et al., 1997), suggesting a more direct role in tau pathology (Su et al., 1992; Snow et al., 2021). Among the different structural features, the 3-*O*-sulfated glucosamine residue have been linked to the HS intracellular accumulation observed in AD, as reproduced in cellular and animal models of tau pathology (Sepulveda-Diaz et al., 2015; Maïza et al., 2018; Huynh et al., 2022). In Human cells, 3-*O*-sulfation is catalyzed by the family of enzymes known as 3-*O*-sulfotransferases (HS3STs) with each producing distinct structural motifs with unique functional properties (Ouidja et al., 2024). Despite the growing evidence of HS involvement in neurodegeneration, the precise contribution of 3-*O*-sulfated heparan sulfates (3S-HS) to tau pathology and their relationship with other disease players remain poorly understood. There is also emerging evidence implicating inflammatory and stress-related signaling pathways in modulating both HS biosynthesis (Parish, 2006; Thacker et al., 2014) and neurodegeneration (Van Horssen et al., 2003). In particular, transforming growth factor beta 1 (TGFβ1) plays a role in both processes (Parish, 2006). TGFβ1 is a pleiotropic cytokine known to orchestrate transcriptional regulation through the activation of Smad-dependent signaling (Nakao et al., 1997) and whose levels are increased in AD brains (Wyss-Coray et al., 1997; Tominaga and Suzuki, 2019). Whereas TGFβ1 signaling has been associated with glial activation, ECM remodeling and neuronal dysfunction (Lesné et al., 2002), HS3ST2 expression has been associated with tau pathology development (Sepulveda-Diaz et al., 2015; Huynh et al., 2022). This opens to the question whether TGFβ1 can impact tauopathy through HS3ST2 expression modulation. Answering this yet unexplored question could help to increase our knowledge of the cellular mechanisms leading to tau pathology in neurons. Here, we used primary hippocampal neurons (PHN) from the rTg4510 mouse model (Ramsden et al., 2005; Han et al., 2012) to investigate whether alterations in the expression of HS and/or 3S-HS biosynthetic enzymes can be regulated by TGFβ1 signaling and whether this contributes to the pathological mechanisms leading to tau pathology. Moreover, we examined the molecular events by which the intracellular 3S-HS and their biosynthetic Hs3st’s can promote tau aggregation and affect synaptic integrity. Finally, we showed the contribution of specific Hs3st’s to the mechanisms leading to tau pathology and to assess their potential as a novel therapeutic target.

## Materials and methods

All products references are listed in Key resources table (Supplementary Table).

### Mouse model

The rTg4510 mice express a repressible form of human tau containing the P301L mutation under the control of a tetracycline–responsive element (TRE), to an activator line expressing a tetracycline-controlled transactivator (tTA) with the CaMKIIα promoter. rTg4510 mice were generated by crossing male C57BL/6 mice expressing the tetracycline-controlled transactivator protein under control of the forebrain-specific calcium-calmodulin-dependent kinase II (CaMKIIα) promoter (CaMKIIα mice) with female FVB mice carrying the responder tetO MAPT*P301L transgene under the control of the Tet-responsive element (tetO) (FVB-*Fgf14*^Tg(tetO–MAPT*P301L)4510Kha^/JlwsJ, also known as FVBTetO-MAPT*P301L). Mice hemizygous for the tetO-MAPT*P301L transgene (±) and for the tetracycline controlled transactivator protein (±) are considered rTg4510 (Ramsden et al., 2005; Han et al., 2012). The non-carrier littermate animals for the tetOe- MAPT*P301L transgene (-/-) and the tetracycline controlled transactivator protein (-/-) were considered as Controls. For all studies, both male and female mice were used and they were allocated randomly between experimental groups. Experimenters were blinded to the genotype of each mouse during data collection and identities were decoded later after analysis.

### Conditioned medium from primary cell culture of rat cortical glial cells

Primary glial cells were prepared from PND5 Sprague-Dawley rats (Janvier Labs). The cortices were dissected and processed as previously described (Maïza et al., 2020). Briefly, the head of PND5 Sprague-Dawly rats was cut to remove the brain. Both hemispheres were recovered and, after removal of the meninges, the hippocampus was removed and both occipital hemispheres were dissected out. The occipital hemispheres were collected, washed twice with HBSS and incubated for 12 min in 2.5% trypsin (Sigma-Aldrich/Merck), and 2.5% DNase (Sigma-Aldrich/Merck) in HBSS at 37°C. Trypsin was inactivated with 10% FBS (Gibco). Cells were plated in 75 cm^2^ flasks in Dulbecco’s Modified Eagle’s Medium (DMEM high glucose GlutaMAX TM-I, Gibco) supplemented with 10% FBS (Gibco) and 0.1% Penicillin-Streptomycin (Life Technologies). Cells were maintained at 37°C in a humidified 5% CO_2_ incubator. When 80% confluence was reached, cells were passaged at 1:2. Passages 1 and 2 were used to obtain the conditioned medium by replacing the DMEM supplemented medium by pre-warmed Neurobasal medium supplemented with 2% B27.

### Primary cell culture of murine hippocampal neurons

Female FVBTetO-MAPT*P301L mice were crossed with a male CaMKIIα-tTA mouse both maintained at the Gly-CRRET animal house. Primary hippocampal cell cultures were prepared from double transgenic E16 embryonic rTg4510 mice. Briefly, the head of embryos was cut to remove the brain. Both hemispheres were recovered and after removal of the meninges, the hippocampus was dissected out in dissection medium (HBSS; Gibco), 0.1% HEPES (Gibco) supplemented with Penicillin-Streptomycin (Life technologies). The hippocampus was collected, washed twice with HBSS, and incubated for 12 min in 2.5% trypsin (Sigma-Aldrich/Merck) and 2.5% DNase (Sigma-Aldrich/Merck) in HBSS at 37°C. Trypsin was inactivated with 10% FBS (Gibco). Cells were dissociated and seeded in 12 well plates coated with poly-D-lysine (Sigma-Aldrich/Merck) or 12 well IBIDI chambers (IBIDI) at a density of 200,000 cells or 35 000 cells, respectively. Cells were seeded in neurobasal medium supplemented with 2% B27 (Life Technologies), GlutaMAX TM-I (Gibco) and 0.1% Penicillin-Streptomycin. Primary cultures were maintained at 37°C in a humidified 5% CO_2_ incubator. After 2 h, the medium was replaced by the glial cell-conditioned medium.

### Transduction of PHN

To transduce primary hippocampal neurons (PHN) in culture with lentivirus we used the Mission Lentiviral Transduction Particles (Sigma-Aldrich/Merck) issued from a library of sequence-verified lentiviral plasmid vectors for sh*Hs3st2*, sh*Hs3st4*, and a shNT (Key resources table). The lentiviral transduction particles were titred via a p24 antigen ELISA assay and pg/mL of p24 were then converted to transducing units per mL using a conversion factor. At day *in vitro* 15 (DIV15), 5 viral particles per cell were added to the PHN and were kept under culture for a further 5 days (DIV20).

### Pharmacological treatment of PHN

To induce or block the activation of the TGFβ1 pathway in PHN we used recombinant human TGFβ1 (Peprotech) at 20 ng/mL or the ALK4, 5 and 7 inhibitor SB431542 (10 μM, Sigma-Aldrich/Merck), which blocks TGFβ signaling, at day *in vitro* 15 (DIV15). DMSO was added as a control using the same volume in which the compounds were solubilized. PHN were kept under culture to DIV20.

### Immunoblotting of RIPA-extracted proteins

The samples were harvested in RIPA buffer (50 mM Tris, pH 8.0, 150 mM NaCl, 0.1% Triton X-100, 0.5% sodium deoxycholate, 0.1% SDS) supplemented with 1% protease inhibitor mixture (Sigma-Aldrich/Merck) and 1% phosphatase inhibitor cocktail I & II (Sigma-Aldrich/Merck). Cell lysates were centrifugated at 15,000 g for 20 min and protein content in supernatants were determined with the BCA Protein Assay kit (Thermo Fisher Scientific) using bovine serum albumin (BSA) as a standard provided in the kit. Cell lysate supernatants containing 1–10 μg of protein were suspended in Laemmli buffer (0.125 M Tris HCl, pH 6.8, 4% SDS, 20% glycerol, 0.004% 9-bromophenol blue; Biorad) supplemented with 10% β-mercaptoethanol and heated for 5 min at 95°C. The proteins were separated on a 8% acrylamide gel and transferred to a PVDF membrane with a Transblot Turbo device (BioRad) for 15 min. The membrane was blocked with a blocking buffer, 5% non-fat milk PBST to prevent non-specific binding. The membranes were incubated with their respective antibodies specific for the target protein overnight at 4°C with gentle agitation. After washing, blots were incubated for 1 h at room temperature with the corresponding secondary antibodies in the blocking buffer. Revelation was performed with Clarity Max ECL Western Blotting Substrates (Bio-Rad) following the manufacturer’s instructions.

### Immunoblotting of high salt sarkosyl oligomers

To isolate tau oligomers, the samples were harvested as previously described (Huynh et al., 2022) in salt-rich sarkosyl buffer (50 mM HEPES pH 7.0, 250 mM sucrose, 1 mM EDTA, 0.5 M NaCl, 1% sarkosyl) supplemented with 1% protease inhibitor mixture (Sigma-Aldrich/Merck) and 1% phosphatase inhibitor cocktail I and II (Sigma-Aldrich/Merck). Lysates were centrifugated at 180,000 g for 30 min and protein content in supernatants was determined using the BCA Protein Assay Kit (Thermo Fisher Scientific). Cell lysate supernatants containing 1–10 μg protein were treated or not (as indicated) in Laemmli buffer (Biorad) supplemented with β-mercaptoethanol. Samples treated with Laemmli buffer were heated for 5 min at 95°C to denature the tau oligomers. The proteins were separated by electrophoresis as described above. Immunoblotting was performed as described above with the antibody T22. Blots were incubated for 1 h at room temperature with the corresponding secondary antibodies in blocking buffer. Revelation was performed with Clarity Max ECL Western Blotting Substrates (Bio-Rad) following the manufacturer’s instructions.

### Immunofluorescence of PHN

Primary neurons maintained in culture for 20 days *in vitro* were rinsed with cold PBS and then fixed at 4°C in cold methanol for 5 min. Cells were rinsed with cold PBS (three times). For immunofluorescence staining, cells were washed with PBS or alternatively with PBS containing 0.01% Triton-x100 (PBS-T) for permeabilization during 15 min at room temperature. Unspecific sites were blocked with PBS (or PBS-T) containing 3% BSA for 60 min at room temperature prior to incubation with primary antibodies (Key resources table). Cells were incubated with the first antibody in blocking solution (3% FBS, PBS-T) overnight at 4°C. Cells were then washed with PBS (or PBST) and incubated with the secondary antibody for 60 min at room temperature. After washing with PBS, nuclei were stained with DAPI (1 μg/mL in PBS) for 5 min and washed with PBS before mounting with Prolong Gold antifade reagent. Stack images were obtained with the software CellSens from a spinning disk inverted confocal microscope (IX81 DSU Olympus, 60 × N.A.1.35) coupled to an Orca Hamamatsu RCCD camera.

### RNA isolation from HPN

RNA was extracted using NucleoSpin RNA XS, Micro kit for RNA purification (Macherey- Nagel) following the manufacturer’s instructions. RNA was eluted in a volume of 15 μL of H_2_O (RNase free) and centrifuged at 1,100 g for 30 s. RNA was either stored at −80°C or directly quantified.

### RNA isolation from hippocampal formation

RNA was extracted using Trizol (Qiazol) followed by the RNeasy mini kit (Qiagen). Hippocampal formation tissue was lysed with a rotor-station homogenizer by addition of Trizol and the tissue was homogenized. Chloroform was added and mixed until a homogeneous solution was formed. Sample were centrifuged at 12,000 g for 10 min. The aqueous phase was transferred to a new microcentrifuge tube. Then, 100% ethanol was added to the super aqueous phase and mixed by pipetting. Samples were then transferred to a RNeasy spin column placed in a 2 mL collection tube following the manufacturer’s instructions. A RNase spin column was placed in a new microcentrifuge tube and 15 μL of RNase free water was added directly to the column and which was centrifuged for 1 min at 8,000 g. RNA was either stored at −80°C or directly quantified.

### RNA expression analysis

RNA (200 ng per sample) was converted to cDNA using the AffinityScript Multiple Temperature cDNA synthesis kit according to the manufacturer’s instructions with the reaction proceeding at 25°C for 10 min, 50°C for 60 min and then 70°C for 15 min. All buffers and solutions used were provided in the kit. Reactions were prepared using 250 nM of upstream primer and 250 nM of downstream primer. Nuclease-free PCR-grade water was added to adjust the final volume to 20 μL. Gene expression was analyzed using the template cDNA by quantitative real time polymerase chain reaction (qPCR) using the Brilliant III Ultra-fast SYBR* kit (Agilent Technologies) in an Agilent AriaMx thermocycler. Analysis was performed with the Agilent AriaMx 1.0 software.

### Histopathological analysis of mice brain with the chromogen DAB

Fixed mouse brain tissue was prepared from free-floating tissue. The brain sections were collected in a washing buffer (PBS) containing 25% Triton X-100. To quench endogenous peroxidase activity, slides were incubated for 10 min in 3% hydrogen peroxide in 20% methanol. After rinsing the slides two times with MilliQ water for 5 min, slides were washed two times more in PBS containing 0.25% Triton X-100 for 10 min and then they were incubated with 5% goat serum (Thermo Fisher Scientific) in PBS for 30 min at room temperature and washed. Slides were then incubated with AT8 primary antibody (Thermo Fisher Scientific, 1:500) in 0.1 M PBS containing 0.25% Triton X-100 overnight. After washing 3 times (5 min each) in PBS containing 0.25% Triton X-100, samples were incubated for 60 min in a secondary goat anti-mouse biotinylated IgG1 in PBS 0.25% Triton X-100. Then, after washing 3 times for 5 min in PBS 0.25% Triton X-100, slides were treated with the Elite ABC kit following the manufacturer’s instructions, rinsed for 10 min with PBS 0.25% Triton X-100 and then 50 mM Tris buffer containing 150 mM NaCl (pH 7.4) twice (10 min each). Slides were developed by a short incubation in 0.1% 3,3′-diaminobenzidine (DAB) peroxidase substrate solution containing 0.05% hydrogen peroxide diluted in Tris buffered-saline and the reaction was stopped by dilution. Slides were mounted in Superfrost Plus slides in 0.3% gelatin diluted in PBS. The day after, slides were rehydrated and dehydrated with increasing ethanol concentrations 30, 70, 90, 100%, and finally with xylene. Sections were scanned with a NanoZoomer (Hamamatsu).

### RNA sequencing and data analysis

Total RNA and library integrity were verified on LabChip Gx Touch 24. Between 197.97–276.9 ng of total RNA were used as input for SMARTer Stranded Total RNA Sample Prep Kit-HI Mammalian (Clontech). Sequencing was performed on the Novaseq 6000 Illumina with S1 cartridge (200 cycles) of 1,600 million reads in Paired ends. Raw reads were visualized by FastQC to determine the quality of the sequencing. Trimming was performed using Trimmomatic with the following parameters LEADING:3 TRAILING:3 SLIDINGWINDOW:4:15 HEADCROP:4, MINLEN:4. High-quality reads were quantified with Salmon. Briefly, a transcriptome index was constructed using a reference genome (GRCm39). Salmon uses an indexing method that allows for rapid alignment-free quantification. For quantification, the processed reads were aligned to the transcriptome index and the software estimated the abundance of transcripts or isoforms. The output of Salmon provides estimated counts or normalized values of transcript abundance. Differential gene expression was done with DESeq2. DESeq2, which normalizes the raw read counts, estimates dispersion to model variability and employs a negative binomial distribution to statistically assess differential expression. It computes *p*-values and fold changes, adjusted for multiple testing using methods like FDR. For Gene ontology (GO) enrichment analysis we used EnrichGO, which takes a list of genes and assessed their enrichments in specific biological processes, molecular functions and cellular components. The tool utilizes statistical methods to compare the input gene list with the GO annotations in a reference database. EnrichGO calculates *p*-values and applies multiple testing corrections to identify significantly enriched GO terms.

### Transcription factor binding site prediction

Transcription factor binding sites (TFBS) for Smad4 were predicted in the promoter regions of mouse Hs3st’s genes using the TFinder web tool (Schultheis et al., 2022). The analysis was performed to investigate potential regulatory mechanisms controlling the expression of genes involved in HS biosynthesis. For sequence retrieval and analysis parameters, target genes included three mouse HS sulfotransferase genes: *Hs3st1*, *Hs3st2*, and *Hs3st4*. Promoter sequences were extracted from the mouse genome assembly GRCm39 using the TFinder platform’s integrated NCBI API functionality. The analysis window was set to ± 2,000 bp from the transcription start site (TSS), encompassing a total region of 4,000 bp per gene to capture core regulatory elements typically found in promoter regions. For motif analysis Smad4 binding sites were predicted using two JASPAR database motifs: MA0925.2 (sma-4): Consensus sequence TGTCTGG MA1153.2 (Smad4): Consensus sequence TGTCTAG. Each motif was analyzed both individually and in combination with each target gene to comprehensively identify potential binding sites. The analysis utilized Position Weight Matrices (PWMs) derived from experimentally validated binding data to model transcription factor-DNA interactions. For statistical parameters the relative score threshold was set to 0.85 to ensure high-confidence binding site predictions. This threshold represents the similarity between candidate sequences and the consensus motif, calculated as the normalized PWM score. Experimental *p*-value calculation was enabled to assess the statistical significance of predicted binding sites, accounting for background nucleotide composition and motif complexity. For data analysis and visualization, results were generated in tabular format containing genomic coordinates, sequence information, scoring metrics and statistical significance values. PWM visualizations were automatically generated for each motif, displaying nucleotide preferences and information content at each position within the binding site.

### Statistical analyses

Statistical analyses were performed using GraphPad Prism software version 10. Values are expressed as the standard error of the mean (SEM). Statistical significance was assessed using the Welch’s *t*-test, which accounts for unequal variances when comparing two groups, or one-way ANOVA with Benjamini, Krieger and Yekutieli *post hoc* analyses for multiple comparisons between a same group of variables or standard deviation (SD), as indicated, considering biological and technical replicates.

## Results

### HS biosynthetic machinery is altered during tau pathology

**FIGURE 1:**
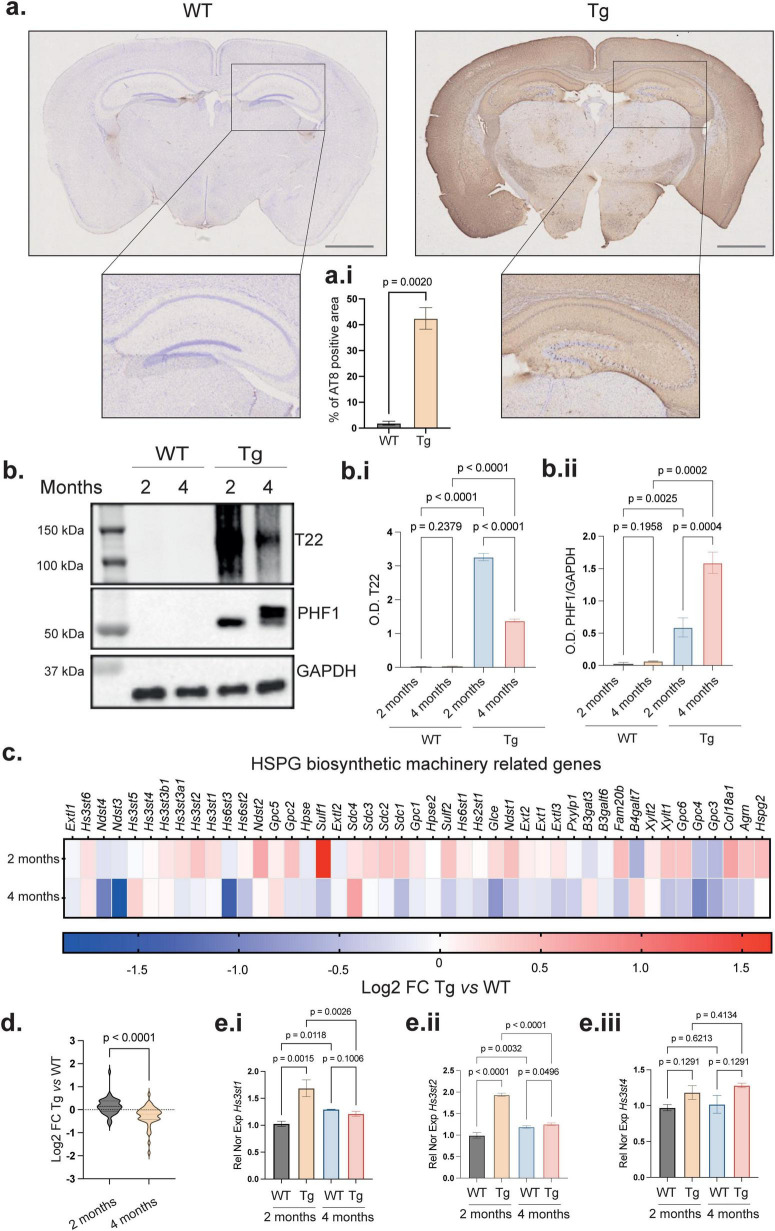
Temporal relationship between tau pathology and HSPG biosynthetic machinery in rTg4510 mice. **(a)** Immunohistochemical detection of abnormally phosphorylated tau (AT8) in free floating sections of Tg and WT mice at 4 months of age. Tg shows a higher AT8 positive area compared with the WT. Nuclei were counterstained with cresyl violet (blue). Scale bar 2 mm. **(a.i)** Quantitative analysis of the AT8-immunoreactive area shows a higher AT8 positive staining area of Tg mice compared with control WT. Error bars represent SEM from 3 mice tissue slides with three fields analyzed by mouse. **(b)** Representative immunoblots of sarkosyl-extracted fraction from hippocampal formation showing elevated levels of soluble tau oligomers (T22) in 2-month-old Tg mice; these levels declined in 4 months-old mice whereas phosphorylation of tau at s396/404 (PHF1) increased with age. **(b.i)** (T22) and **(b.ii)** (PHF1) relative quantification of tau oligomers and tau abnormal phosphorylation normalized with the loading control GAPDH. Error bars represent SEM from 3 mice with two technical replicates. **(c)** Heatmap from RNA-sequencing analysis of hippocampal formation showing upregulation at 2 months of 37 out of 45 genes involved in the HSPG biosynthetic pathway comparing the Log2 FC of Tg vs. WT at 2 and 4 months (3 mice by group). **(d)** Group comparison of differential expressed genes at 2 and 4 months revealing a general decrease in HSPG biosynthesis at 4 months. Error bars represent SEM from 3 mice by group. **(e.i–iii)** qPCR validation of *Hs3st1*, *Hs3st2* and *Hs3st4* gene expression in WT and Tg hippocampal formation at 2 and 4 months. Relative normalized expression with *Rplp0*. Error bars represent SEM from 3 mice transcript extracts; each tested three times. Statistical significance for simple comparison was assessed using Welch’s two-tailed *t*-test for two groups comparisons and one-way ANOVA with Benjamini, Krieger, and Yekutieli *post hoc* analysis for multiple comparisons.

To investigate the relationship between expression of the HS biosynthetic machinery genes and tau pathology, we used the rTg4510 (Tg) mouse model carrying a transgene that expresses the human tau protein with the P301L mutation (Ramsden et al., 2005). This transgenic model exhibits many key features of human tauopathies, including the development of NFTs, neuronal loss and gliosis (Han et al., 2012). As in humans, these pathological changes are observed at early disease stages in the hippocampus, a region of the brain critically involved in cognitive function (Bellenguez et al., 2022). These mice also show behavioral and cognitive deficits that mimic those seen in human tauopathies (Bellenguez et al., 2022). We first validated the presence of tau pathology in this model at 4 months by immunohistochemistry in Tg brain slices using the anti-phosphorylated tau antibody AT8, which recognizes abnormally phosphorylated tau at epitopes Ser202/Thr205. As expected, we confirmed the presence of tau pathology in the forebrain (Figures 1a,a.i) (*p* = 0.0020). Then, evaluated the presence of tau oligomers, which are detectable with the T22 antibody (Lasagna-Reeves et al., 2012) and are characteristically present from the early stages of the disease evolution. With this aim, we recovered brains from Tg and WT mice at 2 and 4 months and dissected the hippocampal formation (HPCF). High salt sarkosyl-fractionation of HPCF homogenates enabled the separation of the soluble oligomers from insoluble advanced aggregates (Huynh et al., 2022). As expected, immunoblotting with T22 did not show any soluble oligomer at 2 and 4 months in HPCF homogenates from the WT animals (Figures 1b,b.i) (*p* = 0.2329 for T22 WT 2 vs. 4 months), whereas comparison of the Tg HPCF homogenates at same ages showed high levels of tau oligomers at 2 months followed by a decrease at 4 months (Figures 1b,b.i) (*p* < 0.0001 for T22 Tg 2 vs. 4 months). This decrease could be due to the evolution of tau to more advanced fibrillation stages, as shown by the increase of phosphorylated tau at Ser396/404 (PHF1) at the molecular weight characteristic of NFTs (Adams et al., 2009) (Figures 1b,b.ii) (*p* = 0.0004 for PHF1 Tg 2 vs. 4 months). Then, to explore whether the expression of the HSPG biosynthetic machinery genes was altered during the progression of AD-related tau pathology, we performed RNA-sequencing from HPC formation of 2- and 4-months old WT and Tg mice. We found an increased expression of 37 out of 45 genes involved in HSPG biosynthesis (Ouidja et al., 2024), including *Ext1*, *Hs6st2*, *Hs6st3*, *Hs3st1*, *Hs3st2*, and *Hs3st4* (Figure 1c), in agreement with previous reports (Sepulveda-Diaz et al., 2015; Huynh et al., 2019; Wang et al., 2023). Interestingly, the highly significant increase in the expression of several genes of the HSPG biosynthetic machinery observed at 2 months was followed by a decrease at 4 months (Figure 1d) (*p* < 0.0001). We confirmed this dynamic expression of *Hs3st1* and *Hs3st2* between 2 and 4 months old Tg mice by performing qPCR of *Hs3st1* (Figure 1e.i) (*p* = 0.0026) and *Hs3st2* (Figure 1e.ii) (*p* < 0.0001), with *Hs3st4* kept stable (Figure 1e.iii) (*p* = 0.4134). These 3 genes have been involved in 3S-HS biosynthesis in the brain and reported to play a role in tau aggregation (Sepulveda-Diaz et al., 2015; Huynh et al., 2019; Wang et al., 2023). Interestingly, this dynamic pattern of Hs3st expression levels correlated with the increased and subsequent decreased tau oligomerization in 2- and 4-months mice and suggested a relationship between the 3-*O*-sulfation of HS chains of HSPGs and the early stages of tau pathology in the HPCF, an area primary affected during neurodegeneration. Notably, when comparing the levels of 3S-HS by IHC in the 2- vs. 4-month-old mice hippocampus, any significant difference was observed (not show) regardless of a reducing gene expression. This is possibly due to the HS accumulation along the disease evolution, as previously reported for HS in human (Su et al., 1992; Goedert et al., 1996; Snow et al., 2021) and for 3S-HS in cell models of tau pathology (Sepulveda-Diaz et al., 2015; Huynh et al., 2022), although future research is required to confirm this eventuality in the mouse model, we thought to focus this study at the neuronal cell level.

### Degenerating primary hippocampal neurons accumulate both 3S-HS and tau

**FIGURE 2:**
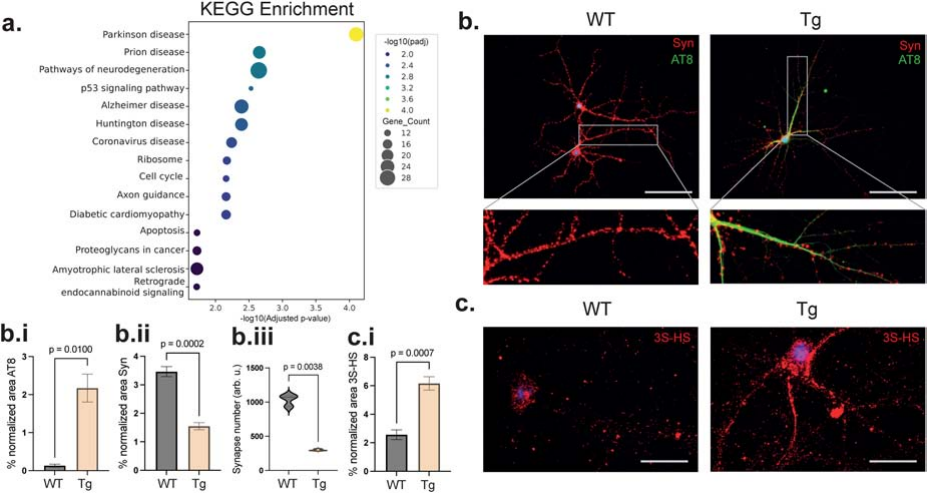
Tg primary hippocampal neurons recapitulate key features of tau-related neurodegeneration. **(a)** Gene ontology (GO) with EnrichGO and KEGG pathway analysis from RNA sequencing comparing PHN from Tg vs. WT (*n* = 3 mice by group) reveals significant enrichment of pathways related to neurodegeneration, including AD, Parkinson disease, Prion diseases and Huntington disease. **(b)** Immunofluorescence staining shows increased tau phosphorylation (AT8-green) in Tg PHN compared to WT. Co-staining with anti-synaptophysin (red) demonstrates reduced synaptophysin levels and decreased synaptic bouton density in Tg PHN. Scale bar represents 50 μm. **(b.i–iii)** Quantification of normalized fluorescence intensity confirms increased AT8 immunoreactivity, decreased synaptophysin protein levels and synapse number. Error bars represent SEM from 3 independent cultures with three analyzed fields by culture. **(c)** Immunofluorescence analysis using HS4C3 (red) antibody, which recognizes 3S-HS, reveals elevated levels of 3S-HS in Tg PHN. Scale bar represents 25 μm. **(c.i)** Quantification of normalized fluorescence intensity confirms elevated 3S-HS in Tg vs. WT PHN (*n* = 3 different cultures each analyzed three times). Statistical significance was assessed using Welch’s two-tailed *t*-test; *p*-values are indicated in the plots. Statistical significance was assessed using Welch’s two-tailed *t*-test.

To investigate whether the 3S-HS accumulation correlated with tau deposition in cultured hippocampal neurons, we developed an *in vitro* neuronal model of tau pathology. Primary hippocampal neurons (PHN) cultures are a widely used experimental model, offering several advantages for studying neuronal processes and neurodegenerative diseases (Tomassoni-Ardori et al., 2020; Tourville et al., 2022). Thus, we dissected PHN from embryonic day 16 (E16) WT and Tg HPC formation as previously described (Maïza et al., 2020). Then, we performed RNA sequencing of WT and Tg PHN, followed by gene ontology (GO) and KEGG pathway analyses of the differentially expressed genes (DEG) using EnrichGO. As expected, the enrichment analysis revealed pathways related to neurodegeneration, with AD and Parkinson’s Disease emerging among the most significantly enriched (Figure 2a). Subsequently, to confirm that cultured neurons from Tg mice express *Hs3st2* and develop tau pathology, we performed immunostaining using an anti-HS3ST2 antibody and the anti-phosphorylated tau antibody AT8 (Supplementary Figures 1a,a.i,a.ii) (*p* = 0.0160 for AT8 WT vs. Tg, *p* = 0.0020 for Hs3st2 WT vs. Tg). Tau pathology was confirmed by immunoblotting using antibodies against three distinct domains of the pathological tau protein. Specifically, we targeted the residues 312–322aa (MC1) in the third microtubule binding domain, the phosphorylated residues Ser202/Thr205 (AT8) in the tau proline rich domain, and residues Ser396/404 (PHF1) within the C-terminus domain. The results showed an increase in all three of these tau phosphorylated residues (Supplementary Figures 1b). Immunofluorescence analysis of neurons co-stained with AT8 and synaptophysin, a marker of synaptic density and function (Song and Augustine, 2015), confirmed the abnormal increase in the phosphorylation of tau (AT8) and reduced synaptophysin protein levels (Figures 2b,b.i,b.ii) (*p* = 0.0100 for AT8, *p* = 0.0002 for % synaptophysin) with fewer synaptic boutons (Figure 2b.iii). (*p* = 0.0038), which exhibited a normal distribution Immunofluorescence using the anti 3S-HS antibody (HS4C3), which predominantly recognizes 3S-carrying sequences in HS (3S-HS) (Sepulveda-Diaz et al., 2015; Huynh et al., 2022), showed an increase in the 3-*O*-sulfated polysaccharide in the Tg PHN compared to WT (Figures 2c,c.i) (*p* = 0.0007). These findings confirm that Tg PHN exhibit the accumulation of 3S-HS together with the deposition of abnormally phosphorylated tau and synaptic loss, key features of AD. Interestingly, the increase in 3S-HS in the diseased cells is in agreement with previous literature suggesting their involvement in the neurodegenerative process (Sepulveda-Diaz et al., 2015; Huynh et al., 2022). However, the molecular players responsible for this increase in cells that develop tau pathology remained unclear.

### TGFB1 controls *Hs3st*s expression and affects tau pathology

**FIGURE 3:**
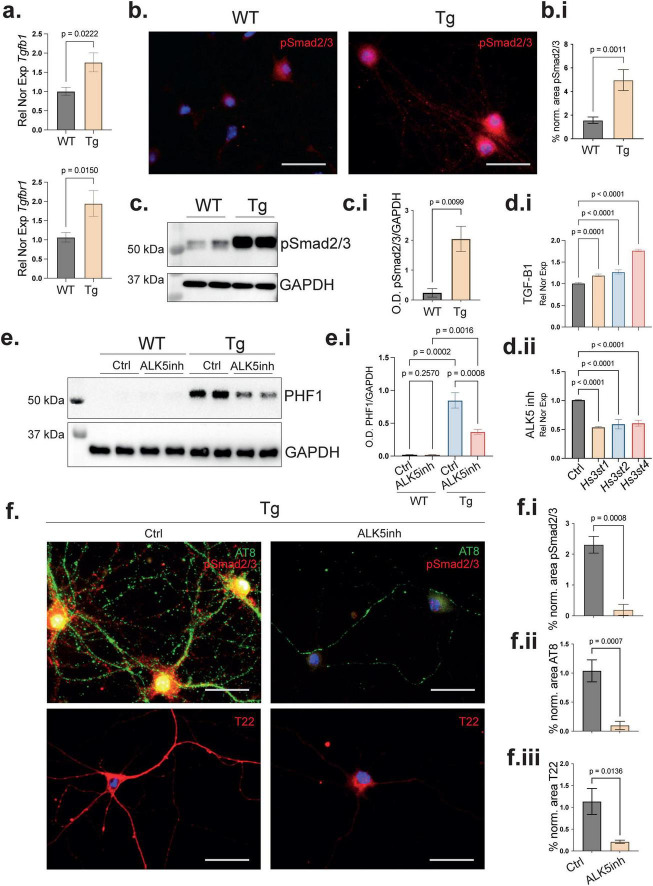
TGFβ1 signaling regulates *Hs3st* gene expression and modulates tau pathology. **(a)** qPCR analysis of PHN from WT and Tg shows increased expression of *Tgfb1* and its receptor *Tgfbr1* in Tg neurons. Relative normalized expression with *Rplp0*. Error bars represent SEM from 3 independent cultures with three replicates by culture. **(b)** Immunofluorescence showing increase phosphorylation of Smad2/3 (pSmad2/3-red) in Tg. pSmad2/3 exhibits nuclear localization in Tg, suggesting active transcription. **(b.i)** Quantification of normalized fluorescence intensity confirms increased pSmad2/3 immunoreactivity in Tg. Error bars represent SEM from 3 independent cultures with three fields analyzed by culture. **(c)** Western blot analysis showing an increase of pSmad2/3 in Tg. **(c.i)** pSmad2/3 quantification of c. normalized with the loading control GAPDH. Error bars represent SEM from 2 independent cultures with two blots analyzed by culture. **(d.i,ii)** Pharmacological modulation of the TGFβ1 pathway reveals that activation with recombinant TGFβ1 increases expression of *Hs3st1*, *Hs3st2*, and *Hs3st4*, while inhibition using an ALK5 (TGFBR1) inhibitor reduces their expression, as shown by qPCR analysis. Relative normalized expression with housekeeping gene *Rplp0*. Error bars represent SEM from 3 independent cultures with three replicates by culture. **(e)** Western blot of tau phosphorylated (PHF1) shows a reduction in tau phosphorylation in Tg treated with the ALK5 inhibitor compared to DMSO controls. **(e.i)** PHF1 quantification normalized with the loading control GAPDH. Error bars represent SEM from 2 independent cultures with two blots analyzed by culture. **(f)** Representative immunofluorescence analysis confirms these findings, showing reduced pSmad2/3 (red) levels and decreased tau phosphorylation (AT8-green) following ALK5 inhibition. Co-staining with the oligomer-specific tau antibody T22 (red) also demonstrates a reduction in tau oligomerization. **(f.i–iii)** Quantification of normalized immunofluorescence shows a significant decreased in pSmad2/3 activation, reduced phosphorylated tau, and diminished tau oligomers in ALK5 inhibitor condition. Error bars represent SEM from 3 independent cultures with three fields analyzed by culture. Statistical significance for simple comparison was assessed using Welch’s two-tailed *t*-test for two groups comparisons and one-way ANOVA with Benjamini, Krieger and Yekutieli *post hoc* analysis for multiple comparisons.

The regulation of *HS3ST*s*/Hs3st*s coding genes has been poorly studied, with some studies suggesting regulation by methylation or by master transcription factors (Danková et al., 2018; Zhang Z. et al., 2018). TGFβ1 and its transcription factor Smad4 are commonly found at elevated levels in AD patient brains, (Nativio et al., 2020) suggesting their involvement in the transcription regulation of other genes involved in neurodegenerative processes, such as *Hs3st*s. To explore this possibility, we performed qPCR analysis of WT and Tg PHN transcripts. The results showed increased expression of *Tgfb1* and *Tgfbr1* in the presence of the disease (Figure 3a) (*p* = 0.0222 for *Tgfb1*, *p* = 0.0150 for *Tgfbr1*). The activation of the TGFβ1 signaling pathway was confirmed by fluorescence of phosphorylated Smad2/3 (pSmad2/3), a marker of TGFβ1 pathway activation (Nakao et al., 1997; Figures 3b,b.i) (*p* = 0.0011). Accordingly, pSmad2/3 immunofluorescence revealed a nuclear localization pattern in Tg PHN, suggesting a role in the regulation of gene expression (Figures 3b,b.i). Accordingly, immunoblotting showed increased pSmad2/3 in the Tg cells (Figures 3c,c.i) (*p* = 0.0099). To determine whether selective modulation of the TGFβ1 signaling influenced the expression of *Hs3st* coding genes, we manipulated the pathway using either recombinant TGFβ1 protein as a stimulus or an ALK5 inhibitor to block TGFBR1. Notably, our results indicate that *Hs3st* gene expression was regulated by TGFβ1: the activation of the pathway increased *Hs3st* expression (Figure 3d.i). (*p* = 0.0001 for TGFβ1 effect on *Hs3st1*, *p* < 0.0001 for TGFβ1 effect on *Hs3st2*, *p* < 0.0001 for TGFβ1 effect on *Hs3st4*), while its inhibition resulted in decreased expression (Figure 3d.ii) (*p* < 0.0001 for TGFβ1 inhibitor effect on *Hs3st1*, *p* < 0.0001 for TGFβ inhibitor effect on *Hs3st2*, *p* < 0.0001 for TGFβ inhibitor effect on *Hs3st4*). These results were supported by *in silico* prediction of (Minniti et al., 2025) Smad4 transcription factor binding sites in the promoter region of *Hs3st1*, *Hs3st2*, and *Hs3st4* (Supplementary Figure 2). These results suggest that TGFβ1 induces upregulation of *Hs3st2*. Notably, the TGFβ1 used here was exogenous, whereas under pathophysiologic conditions it can be produced by endogenous sources, including astrocytes. Because it has been previously shown that the modulation of *Hs3st2* expression affects tau pathology (Sepulveda-Diaz et al., 2015; Huynh et al., 2022), we investigated whether inhibition of the TGFβ1 pathway affected the extent of tau pathology in the cultured PHN. Immunoblotting of tau phosphorylated (PHF1) in Tg PHN treated with ALK5 inhibitor compared to DMSO (control) showed a significant decrease in abnormal tau phosphorylation (PHF1) (Figures 3e,e.i) (*p* = 0.0008). This was confirmed by immunofluorescence showing a decrease in pSmad2/3 (Figures 3f,f.i) (*p* = 0.0008) as well as a remarkable reduction in abnormal tau phosphorylation (AT8) (Figures 3f,f.ii) (*p* = 0.0007). Accordingly, immunolabeling with the anti-oligomeric tau antibody T22 showed a decrease in the level of tau oligomers in ALK5 inhibitor-treated PHN (Figures 3f,f.iii) (*p* = 0.0136). Based on these results, we hypothesized that the reduction in tau pathology observed in ALK5 inhibitor-treated neurons could be due to a downstream decrease of *Hs3st*s expression, as suggested for *Hs3st2* in previous reports (Sepulveda-Diaz et al., 2015). However, it remained unclear whether all neural *Hs3st*s are associated with this effect.

### *Hs3st2* loss of function decreases 3S-HS and tau pathology

**FIGURE 4:**
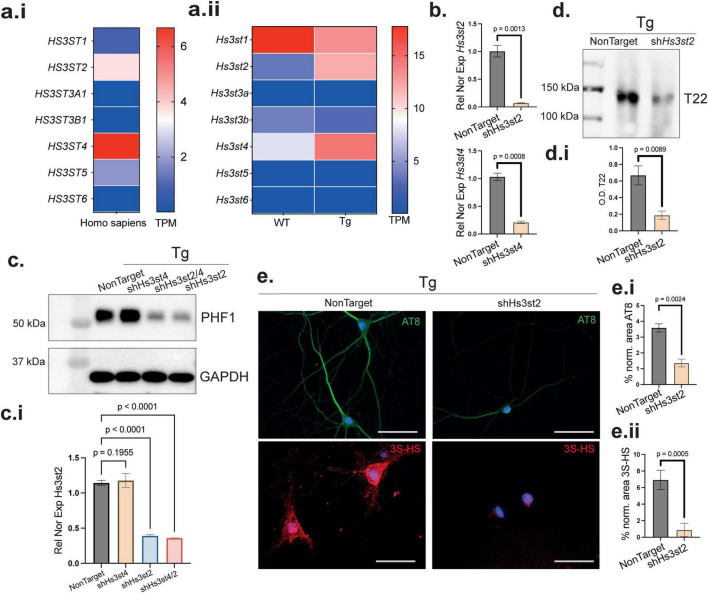
Selective knockdown of *Hs3st2* reduces tau abnormal phosphorylation and oligomerization via 3S-HS. **(a.i)** Heatmap analysis of publicly available human hippocampal transcriptomic data (GTEx) reveals *Hs3st2* and *Hs3st4* as the most highly expressed Hs3st genes in healthy individuals. Error bars represents SD from 3 independent cultures. **(a.ii)** Heatmap analysis from RNA-sequencing of WT and Tg PHN shows that *Hs3st1*, *Hs3st2*, and *Hs3st4* are the most expressed isoforms in WT PHN, with *Hs3st2* and *Hs3st4* significantly upregulated in Tg PHN (*n* = 3). **(b)** Lentiviral-mediated knockdown (shRNA) of *Hs3st2* and *Hs3st4* in Tg PHN results in robust gene silencing, as confirmed by qPCR. Relative normalized expression with *Rplp0.* Error bars represent SEM from 3 independent cultures with three replicates by culture. **(c)** Western blot analysis reveals that knockdown of *Hs3st2*, either alone or in combination with *Hs3st4*, significantly reduces tau abnormal phosphorylation (PHF1), whereas *Hs3st4* knockdown alone has no effect. **(c.i)** PHF1 quantification normalized with the loading control GAPDH. Error bars represent SEM from 3 independent cultures and two technical replicates. **(d)** Representative immunoblotting of Sarkosyl-fractionated soluble protein extracts show decreased tau oligomers (T22) following *Hs3st2* knockdown, indicating reduction in tau soluble oligomers in Tg PHN. **(d.i)** T22 signal quantification normalized by equal protein load. Error bars represent SEM from 3 independent cultures and two technical replicates. **(e)** Immunofluorescence confirms the reduction in phosphorylated tau (AT8-green) and of 3S-HS (HS4C3-red) in *Hs3st2*-LOF PHN. **(e.i,ii)** Quantification of normalized fluorescence intensity confirms a significant reduction in AT8 and HS4C3 immunoreactivity in Tg. Error bars represent SEM from 3 independent cultures with three fields analyzed by culture. Statistical significance for simple comparison was assessed using Welch’s two-tailed *t*-test for two groups comparisons and one-way ANOVA with Benjamini, Krieger and Yekutieli *post hoc* analysis for multiple comparisons.

HS carrying 3-*O*-sulfation (3S-HS) are a specialized subset of HS implicated in several biological processes (Huynh et al., 2019) and involved in the development of tau pathology (Maïza et al., 2018). In humans, seven HS 3-*O*-sulfotransferases are encoded by *HS3ST1*, *HS3ST2*, *HS3ST3a*, *HS3ST3b*, *HS3ST4*, *HS3ST5*, and *HS3ST6* (Maïza et al., 2018). Each one exhibits unique substrate specificity and shows specific expression in different tissues (Maïza et al., 2018; Ouidja et al., 2024). Here, we first investigated their expression in the human HPC by analyzing publicly available data from the Genotype-Tissue Expression (GTEx) project. We found that *HS3ST2* and *HS3ST4* were the *HS3ST* genes with the highest expression in hippocampal tissue from healthy individuals (Figure 4a.i). We then performed RNAseq analysis in our murine WT and Tg PHN. Of the *Hs3st* family members, *Hs3st1*, *Hs3st2*, and *Hs3st4* were the most highly expressed in WT PHN. Only *Hs3st2* and *Hs3st4* were found to be upregulated in Tg PHN (Figure 4a.ii). These findings suggested a specific role for *Hs3st2* and *Hs3st4* in hippocampal function, supporting their involvement in processes leading to AD-related tau pathology (Sepulveda-Diaz et al., 2015; Huynh et al., 2022). Thus, to investigate whether these two genes, *Hs3st2* and *Hs3st4* or possibly only one of them, are involved in the process leading to tau pathology, we performed loss of function (LOF) experiments on each of the two enzymes. We transduced lentivirus containing a shRNA for *Hs3st2* and *Hs3st4*, a combination of both, or a non-target control. qPCR analysis showed high efficiency of both lentiviral transduction and gene silencing for the sh*Hs3st2* and sh*Hs3st4* constructs (Figure 4b) (*p* = 0.0013 for sh*Hs3st2*, *p* = 0.0008 for sh*Hs3st4*). Immunoblotting analysis using the PHF1 antibody in Tg PHN showed that the LOF of *Hs3st4* did not decrease the levels of abnormally phosphorylated tau (Figures 4c,c.i) (*p* = 0.1955). However, *Hs3st2* LOF either alone or in combination with *Hs3st4* LOF resulted in a highly significant decrease in abnormal tau hyperphosphorylation (Figures 4c,c.i) (*p* < 0.0001 for sh*Hs3st2*, *p* < 0.0001 for sh*Hs3st2/4*). Furthermore, immunoblotting of the high salt sarkosyl-fractionation (Huynh et al., 2022) of protein extracts containing tau oligomers revealed a decrease in tau oligomerization, as detected by the T22 antibody after *Hs3st2* LOF (Figures 4d,d.i) (*p* = 0.0089 for sh*Hs3st2*). Immunostaining using the AT8 antibody confirmed the effect of *Hs3st2* LOF on decreasing the abnormal hyperphosphorylation of tau (Figures 4e,e.i) (*p* = 0.0024 for sh*Hs3st2*). Moreover, *Hs3st2* LOF in Tg PHN reduced 3S-HS levels (Figures 4e,e.ii) (*p* = 0.0005 for sh*Hs3st2*), indicating that the Hs3st2 enzyme is active and generates the 3S-HS product within the context of tau pathology. Because these phenotypes were obtained using a single shRNA against *Hs3st2*, off-target effects cannot be fully excluded; therefor using an independent shRNA and/or orthogonal biochemical/protein-level readouts will be important in future work. Altogether, these findings support the involvement of Hs3st2 in AD-related tau pathology in murine neurons. However, it remained unknown whether *Hs3st2* LOF can mitigate defects in synaptic homeostasis induced by tau pathology (Ballatore et al., 2007).

### *Hs3st2* loss of function reduces synaptic alterations

**FIGURE 5:**
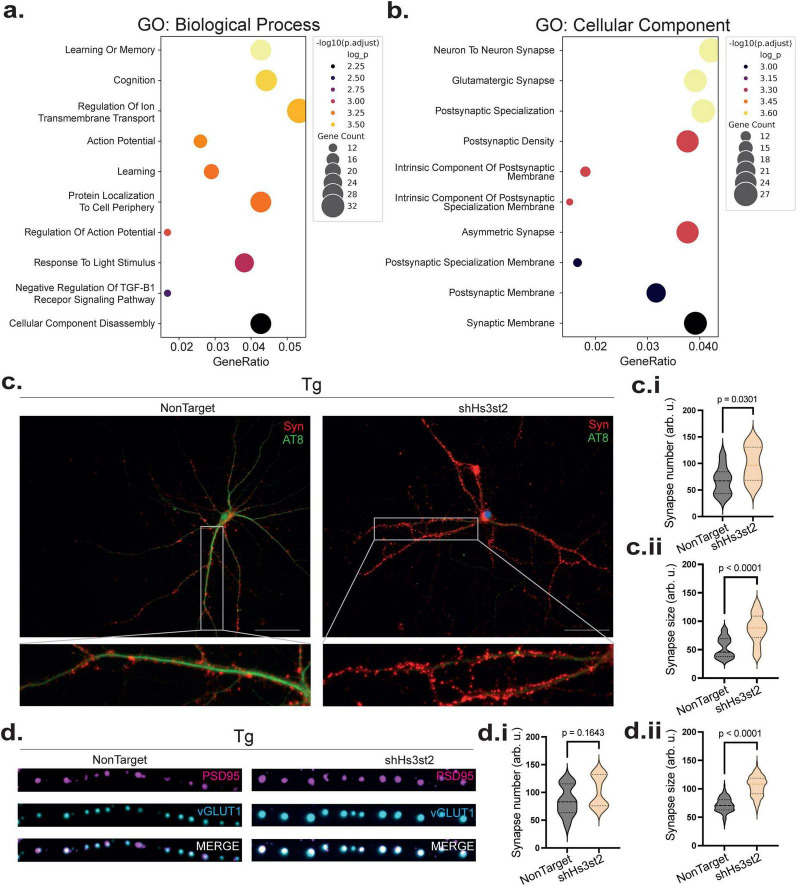
*Hs3st2* knockdown enhances synapse and reverses tau induced synaptic impairment. **(a)** Differential expression and GO enrichment analysis after DGE between Tg control and Tg *Hs3st2* LOF PHN (*n* = 3 by group) revealed a cluster of upregulated genes in the “Biological process” term, genes are associated with cognition, learning, and memory processes. **(b)** Enrichment in “Cellular Component” term also suggests improvement of synaptic components, including postsynaptic specialization and postsynaptic density (*n* = 3 by group). **(c)** Representative co-immunostaining with AT8 (green) and Syn (red), showing a reduction in abnormally phosphorylated tau (AT8) and an increase in synaptic size and number (Syn) after *Hs3st2* LOF. **(c.i,ii)** Quantification of normalized fluorescence intensity shows a significant decrease in AT8 immunoreactivity, increase in synaptophysin protein levels and synapse number in Tg *Hs3st2* LOF in PHN compared to Tg4510 Non-Target (control). Error bars represent SEM from 3 independent cultures with three fields analyzed by culture. **(d)** Co-labeling with the presynaptic marker Vglut-1 and the postsynaptic marker PSD95 revealed increased synaptic size, density and colocalization in Tg *Hs3st2* LOF. **(d.i,.ii)** Quantification of co-labeled fluorescent puncta shows a significant increase in synaptic size in Tg4510 *Hs3st2* LOF PHN compared to rTg4510 control. Error bars represent SEM from 3 independent cultures with three fields analyzed by culture. Statistical significance was assessed using Welch’s two-tailed *t*-test.

The presence of pathological tau at synapses correlates with synaptic impairment in AD (Robbins et al., 2021). Having shown that Hs3st2 is involved in the process leading to tau pathology in PHN we decided to investigate its role at the synapse. Transcriptomic analysis using EnrichGO after the *Hs3st2* LOF in Tg PHN revealed a cluster of upregulated genes for which enrichment analysis showed, at the top of the “Biological process,” genes associated with cognition and learning or memory (Figure 5a). Furthermore, analysis of the genes involved in “Cellular component,” revealed that most of these processes were associated with synapses, especially with postsynaptic specialization and postsynaptic density (Figure 5b). Therefore, we decided to investigate the effect of *Hs3st2* LOF on synapses in the Tg PHN. Co-immunostaining using the AT8 and Syn antibodies showed that *Hs3st2* LOF resulted in an increase in synapse size and number correlating with a decrease in the abnormal phosphorylation of tau (Figures 5c,c.i) (*p* = 0.0301) (Figure 5c.ii) (*p* < 0.0001). Furthermore, co-staining with the presynaptic marker Vesicular glutamate transporter 1 (Vglut1) and with the postsynaptic marker postsynaptic density protein 95 (PSD95) revealed that *Hs3st2* LOF in Tg PHN led to a significant recovery of synaptic size as indicated by colocalization of both markers, suggesting the presence of active synapses (Figures 5d,d.i) (*p* = 0.1643) (Figure 5d.ii) (*p* < 0.0001 for size). Together, these results demonstrated that *Hs3s2 LOF* can restore synaptic homeostasis and enhance synaptic connectivity in this Tg AD-related model of tau pathology.

## Discussion

This work sheds new light on the mechanisms by which 3S-HS chains in HSPGs may contribute in the development and evolution of tau pathology in the hippocampus, a region that is critically affected in neurodegeneration (Huynh et al., 2019). While the role of HSPGs in facilitating tau aggregation is well recognized, our study reveals an upregulation of specific HSPG/HS biosynthetic genes at the early stage of the disease, preceding the accumulation of insoluble tau aggregates in the HPCF (Goedert et al., 1996; Sepulveda-Diaz et al., 2015; Huynh et al., 2022). After first confirming the presence of tau pathology in the hippocampus of the Tg mice, we identified a peak in tau oligomerization at 2 months—an early stage of pathology—followed by a decrease at 4 months. This early rise in tau oligomers was found to correlate with significant upregulation of HSPG biosynthetic genes, remarkably including those involved in the synthesis of 3S-HS: *Hs3st1*, *Hs3st2*, and *Hs3st4*, suggesting that these genes may contribute to the formation or stabilization of early tau oligomers.Interestingly, these genes have been associated with neurodegenerative diseases such as AD (Sepulveda-Diaz et al., 2015; Huynh et al., 2019; Wang et al., 2023). Because tau oligomers appear at early stages of the disease and evolve into aggregates as the disease advances (Lasagna-Reeves et al., 2012), we wondered whether the expression of 3S-HS biosynthetic genes dynamically evolves following either tau oligomerization or tau aggregation. Thus, we developed a controlled *in vitro* setting using cultured PHN derived from Tg mice. We showed that this model offers a unique *in vitro* platform capable of retaining the tau pathology phenotype characteristic of this *in vivo* disease model. Our transcriptomic analysis further supported the relevance of this model for studying AD-related neurodegeneration, as shown by the Gene ontology (GO) and KEGG (EnrichGO) analyses, which reveal a significant enrichment of pathways involved in neurodegenerative processes (Arima et al., 1999; Rawat et al., 2022; Lepinay and Cicchetti, 2023). These results reinforce the relevance of this Tg model for dissecting key mechanisms of tau-driven neurodegeneration, as supported by our results that confirm the high levels of abnormal tau phosphorylation at multiple disease-relevant epitopes (Ser202/Thr205, Ser396/404, and 312–322aa) and by the pronounced reduction in synaptophysin levels and synaptic bouton density in Tg PHN, indicative of synaptic dysfunction—a key feature of early AD (Robbins et al., 2021).

Our new PHN platform allowed us to study the implication of 3S-HS in the AD-related tau pathology in mammalian neurons. The cell-autonomous changes in 3S-HS levels during early tau pathology have previously been poorly defined. In addressing this question, we showed an increase in 3S-HS levels in Tg PHN compared to their WT controls. This indicates that elevated 3S-HS in a genetically driven tau pathology model directly correlates with abnormal tau phosphorylation and synaptic dysfunction at the cellular level. However, since the precise molecular players driving the upregulation of 3S-HS in AD-related tau pathology remained unclear, we turned our attention to exploring specific signaling pathways that could be involved. With this aim, we considered previous reports proposing the involvement of epigenetic mechanisms or transcription factors in these processes (Danková et al., 2018; Zhang Z. et al., 2018). We identified the TGFβ1 signaling pathway as a dynamic regulator of neuronal *Hs3st* genes expression. Using PHN from the Tg mice, we demonstrate that components of the TGFβ1 pathway—including *Tgfb1*, *Tgfbr1*, and phosphorylated Smad2/3—are upregulated under tau pathology conditions, which is consistent with the inflammatory responses previously reported in AD brains (Lesné et al., 2002; Tominaga and Suzuki, 2019; Nativio et al., 2020). Importantly, the selective modulation of the TGFβ1 pathway revealed a functional link between this signaling axis and *Hs3st* expression: treating neurons with exogenous TGFβ1 increased *Hs3st*s transcription, while pharmacological inhibition *via* ALK5 blockade reduced it. This regulatory relationship provides an insight into the causal connection between TGFβ1 activity and *Hs3st* gene expression in humans, whatever the source of TGFβ1 is, neurons, microglia astrocytes or other glial cells. However, while our *in vivo* data are consistent with early engagement of an HS remodeling program during tau oligomerization, the full TGFβ1–pSmad2/3–Hs3st2–3S-HS axis is mechanistically established *in vitro* and inferred (but not directly proven) *in vivo* at similar stage. Future work *in vivo* should explicitly test tissue-level activation of this pathway to connect signaling, enzyme induction, and HS structural outcomes. Concerning tau pathology, inhibition of the TGFβ1 receptor resulted in a marked decrease in tau phosphorylation at disease-relevant epitopes (Ser396/404), as well as a reduction in tau oligomerization. These effects are likely to be mediated, at least in part, by the downstream downregulation of Hs3st enzymes (Sepulveda-Diaz et al., 2015). These findings uncover a new TGFβ1–Hs3st–tau axis, positioning TGFβ1 signaling as a potential upstream driver or enhancer of 3S-HS–mediated tau pathology. However, although our data showed that TGFβ1 signaling can promote tau pathology through the upregulation of *Hs3st* and their 3S-HS product, it remained to be determined whether this is a specific Hs3st regulatory mechanism or a broader consequence of reduced sulfation across multiple Hs3st. Indeed, 3S-HS are produced through the enzymatic activity of multiple Hs3st, and the relative contribution of each enzyme to the generation of 3S-HS species that lead to tau pathology in neurodegeneration or in our PHN is unclear. In humans, the seven *HS3ST* genes (*HS3ST1*–*6*) that encode 3S-HS biosynthetic enzymes show distinct and sometimes overlapping pattern of expression in different cell types and tissues (Maïza et al., 2018). This suggests the existence of specific 3S-HS structures with as yet unknown sulfation patterns and biological functions that are difficult to study (Ouidja et al., 2024). The analysis of GTEx transcriptomic data revealed that *HS3ST2* and *HS3ST4* are the predominant *HS3ST*s expressed in the human hippocampus, suggesting a central role in 3S-HS biosynthesis in this brain region. Supporting this, *Hs3st2* and *Hs3st4* were also found to be expressed in WT PHN and upregulated in the Tg PHN. This upregulation supports the involvement of these specific enzymes in the mechanism leading to AD-related tau pathology. In our Tg PHN model, *Hs3st2* LOF, but not *Hs3st4* LOF, was associated with reduced abnormal tau phosphorylation and decreased tau oligomeric species, together with reduced 3S-HS immunoreactivity. These observations are consistent with a functional link between Hs3st2-dependent 3S-HS biosynthesis and tau-related phenotypes in hippocampal neurons. Additionally, the absence of significant tau modulation following *Hs3st4* LOF suggests that *Hs3st4* upregulation may be compensatory but not functional in this model. However, because our LOF experiments relied on a single shRNA targeting experiment supporting Hs3st2 as a promising regulator of tau pathology, confirming these findings using independent shRNAs and complementary readouts will be important to strengthen causality. Our findings are the first to demonstrate in mammalian neurons that Hs3st2 is a critical regulator of AD-related tau pathology and to propose a mechanistic link between 3S-HS biosynthesis, the TGFβ1, and the process of tau aggregation in hippocampal neurons. Moreover, this indicates that in the tau-related pathology, the modulation of the TGFβ1-Hs3st2-tau axis might mitigate these effects without directly targeting tau, despite the presence of the pathologic mutation. Thus, while *Hs3st2* LOF clearly reduced pathological tau species, we aimed to explore whether this molecular effect could be translated into the benefits for synaptic features.

The accumulation of pathological tau species at synapses is a hallmark of AD and is strongly correlated with synaptic dysfunction and cognitive decline (Robbins et al., 2021). Having established the direct role of Hs3st2 in modulating abnormal tau phosphorylation and aggregation in hippocampal neurons, we next investigated whether this enzyme also contributes to synaptic dysfunction. LOF of *Hs3st2* in the Tg PHN led to significant transcriptional changes, notably the upregulation of gene clusters involved in learning, memory and cognitive function. This agrees with a rescuing effect at the synaptic level. Gene ontology analysis supported this, revealing that among the genes upregulated following *Hs3st2* LOF, those associated with key synaptic structures and processes, including the postsynaptic density, postsynaptic specialization, and neuron-to-neuron synaptic connections, were highly enriched. Complementing these findings, immunohistochemical analysis revealed an increase in synapse number following *Hs3st2* LOF, as indicated by elevated synaptophysin levels. Moreover, the recovery of functional synaptic architecture was confirmed by increased expression and colocalization of Vglut1 (a presynaptic marker) and PSD95 (a postsynaptic marker), in agreement with previous reports on enhanced synaptic connectivity and active neurotransmission (Siano et al., 2019; Bulovaite et al., 2022). Importantly, this synaptic rescue occurred with a concomitant reduction in tau pathology, implying a causal link between 3S-HS biosynthesis, tau accumulation, and synaptic integrity. The ability of *Hs3st2* LOF to reverse synaptic pathology is remarkable, given that synaptic loss is the strongest pathological correlate of cognitive decline in AD (Arriagada et al., 1992). By rescuing synaptic size and connectivity, HS3ST2 inhibition may hold therapeutic potential beyond simply halting disease progression, as it may enable the functional recovery of neuronal circuits. These results point to 3S-HS as possible modulators of synaptic function (Maïza et al., 2020) and raises the possibility that Hs3st2-specific 3S-HS sequences are involved in synaptic signaling, plasticity or structural maintenance in cultured neurons from the rTg4510 mouse. However, while rTg4510 has been highly informative for studying tau-driven neurodegeneration, interpretation and generalizability require caution. The model relies on high expression of mutant human tau (P301L) driven by a tetracycline-responsive system, and tau overexpression itself can amplify phenotypes related to human sporadic disease. In addition, this model is affected by well-described transgene insertion/deletion (“TgINDEL”) effects, including a tau transgene insertion associated with a large deletion at the *Fgf14* locus and additional tTA transgene–related insertional effects that can influence phenotypes independent of tau. Moreover, tTA expression can itself produce strain-dependent hippocampal alterations in some contexts (Ramsden et al., 2005). Accordingly, we interpret our findings as mechanistic insights within a tau overexpression-based experimental platform and emphasize the importance of future validation across complementary models and human datasets.

Fundamentally, although HS is classically viewed as predominantly extracellular, HSPGs/HS species can be internalized (Schultheis et al., 2022), creating intracellular HS pools that traffic through endosomal/lysosomal pathways. Given that endolysosomal trafficking is frequently perturbed in tauopathy, we proposed a mechanism in which a shift in 3S-HS distribution toward intracellular compartments favors encounters between 3S-HS and tau, promoting tau phosphorylation/oligomerization. In line with this interpretation, Sepulveda-Diaz et al. (2015) reported that 3S-HS can be internalized and interact with tau inside cells, and that 3S-HS binds tau rather than key kinases/phosphatases (e.g., GSK3β, PKA, PP2A), supporting a mechanism primarily driven by direct 3S-HS–tau interaction. Here, we give evidence that TGFβ1-can induce the expression of Hs3st2, which product engages tau and promotes tau pathology.

In summary, our study revealed a novel TGFβ1–Hs3st2–tau axis, supporting the hypothesis that 3S-HS synthetized by the enzyme Hs3st2 are key mediators of tau pathology and synaptic dysfunction in a cellular model of AD-related tau pathology. Moreover, our data suggest that, within this TGFβ1–Hs3st2–tau axis, neuroinflammatory signaling can promote tau pathology *via* upregulation of 3S-HS biosynthesis with downstream consequences for synaptic integrity. This represents a shift from viewing TGFβ1 solely as a marker of neuroinflammation to a modifiable driver of tau pathology through HSPG biology. Targeting the upstream TGFβ1–Hs3st2–tau axis may therefore represent a complementary strategy to mitigate tau-driven synaptic dysfunction without directly targeting tau itself, opening new avenues for targeting upstream mechanisms in neurodegenerative diseases, such as AD.

## Data Availability

RNA-seq data have been deposited in public repositories: Mouse tissue RNA-seq data have been submitted to ArrayExpress under accession E-MTAB-16551, https://www.ebi.ac.uk/arrayexpress/experiments/E-MTAB-16551/. Primary hippocampal neuron RNA-seq data are available at NCBI under BioProject accession PRJNA1135370, https://www.ncbi.nlm.nih.gov/bioproject/PRJNA1135370.

## Supplementary material

Supplementary Figure 1 Primary hippocampal neurons exhibit aberrant tau phosphorylation at multiple residues and increased Hs3st2 protein expression. **(a)** Co-Immunofluorescence staining show increased Hs3st2 protein levels (green) and increased abnormal phosphorylation of tau (AT8) in Tg PHN compared to WT. **(a.i,a.ii)** Quantification of normalized fluorescence intensity confirms a significant increase in Hs3st2 and AT8 immunoreactivity. Error bars represent SEM from 3 independent cultures with 3 fields analyzed by culture. **(b)** Western blot analysis shows an increased abnormal phosphorylation of tau (MCI, AT8, and PHF1) in Tg PHN compared with WT. **(b.i–b.iii)** MCI, AT8 and PHF1 quantification of tau abnormal phosphorylation normalized with the loading control GAPDH. Error bars represent SEM from 3 independent cultures analyzed twice. Statistical significance was assessed using Welch’s two-tailed *t*-test.

Supplementary Figure 2 Transcription factor binding site prediction of Smad4 in the promoter region of *Hs3st1*, *Hs3st2*, and *Hs3st4*. **(a)** Transcription factor binding sites (TFBS) for Smad4 were predicted in the promoter regions of mouse *Hs3st1*, *Hs3st2* and *Hs3st4* genes using the TFinder web tool. Position weight matrices (PWMs) for Smad-binding elements (SBEs) were retrieved from the JASPAR database, reconstructed and subsequently employed to scan the promoter regions of *Hs3st1*, *Hs3st2*, and *Hs3st4* for putative Smad4 transcription factor binding sites. **(b)** Sequence Retrieval and Analysis Parameters Target genes included three mouse HS sulfotransferase genes: *Hs3st1*, *Hs3st2*, and *Hs3st4*. The relative score threshold was set to 0.85 to ensure high-confidence binding site predictions. **(c)** Integrative genome viewer and TFBSPred Analysis. Genomic view of chromosome 16 (hg38) showing the *Hs3st1*, *Hs3st2*, *Hs3st4* gene loci, transcription start sites (TSS), and annotated SBEs.

Supplementary Table 1 Key resources table.

## References

[B1] Adams S. J. Crook R. J. Deture M. Randle S. J. Innes A. E. Yu X. Z. (2009). Overexpression of wild-type murine tau results in progressive tauopathy and neurodegeneration. *Am. J. Pathol*. 175 1598–1609. 10.2353/ajpath.2009.090462 19717642 PMC2751556

[B2] Arima K. Hirai S. Sunohara N. Aoto K. Izumiyama Y. Uéda K. (1999). Cellular co-localization of phosphorylated tau- and NACP/alpha-synuclein-epitopes in lewy bodies in sporadic Parkinson’s disease and in dementia with Lewy bodies. *Brain Res*. 843 53–61. 10.1016/s0006-8993(99)01848-x 10528110

[B3] Arriagada P. V. Growdon J. H. Hedley-Whyte E. T. Hyman B. T. (1992). Neurofibrillary tangles but not senile plaques parallel duration and severity of Alzheimer’s disease. *Neurology* 42(3 Pt 1), 631–639. 10.1212/wnl.42.3.631 1549228

[B4] Ballatore C. Lee V. M.-Y. Trojanowski J. Q. (2007). Tau-mediated neurodegeneration in Alzheimer’s disease and related disorders. *Nat. Rev. Neurosci.* 8 663–672. 10.1038/nrn2194 17684513

[B5] Bellenguez C. Küçükali F. Jansen I. E. Kleineidam L. Moreno-Grau S. Amin N. (2022). New insights into the genetic etiology of Alzheimer’s disease and related dementias. *Nat. Genet*. 54 412–436. 10.1038/s41588-022-01024-z 35379992 PMC9005347

[B6] Blair L. J. Nordhues B. A. Hill S. E. Scaglione K. M. O’Leary J. C. Fontaine S. N. (2013). Accelerated neurodegeneration through chaperone-mediated oligomerization of tau. *J. Clin. Invest*. 123 4158–4169. 10.1172/JCI69003 23999428 PMC3784538

[B7] Bulovaite E. Qiu Z. Kratschke M. Zgraj A. Fricker D. G. Tuck E. J. (2022). A brain atlas of synapse protein lifetime across the mouse lifespan. *Neuron* 110 4057–4073.e8. 10.1016/j.neuron.2022.09.009. 36202095 PMC9789179

[B8] Cummings J. L. (2004). Alzheimer’s disease. *N. Engl. J. Med*. 351 56–67. 10.1056/NEJMra040223 15229308

[B9] Danková Z. Braný D. Dvorská D. Ňachajová M. Fiolka R. Grendár M. (2018). Methylation status of KLF4 and HS3ST2 genes as predictors of endometrial cancer and hyperplastic endometrial lesions. *Int. J. Mol. Med*. 42 3318–3328. 10.3892/ijmm.2018.3872 30221668 PMC6202087

[B10] Goedert M. Jakes R. Spillantini M. G. Hasegawa M. Smith M. J. Crowther R. A. (1996). Assembly of microtubule-associated protein tau into Alzheimer-like filaments induced by sulphated glycosaminoglycans. *Nature* 383 550–553. 10.1038/383550a0 8849730

[B11] Han H. J. Allen C. C. Buchovecky C. M. Yetman M. J. Born H. A. Marin M. A. (2012). Strain background influences neurotoxicity and behavioral abnormalities in mice expressing the tetracycline transactivator. *J. Neurosci*. 32 10574–10586. 10.1523/JNEUROSCI.0893-12.2012 22855807 PMC3431916

[B12] Hasegawa M. Crowther R. A. Jakes R. Goedert M. (1997). Alzheimer-like changes in microtubule-associated protein tau induced by sulfated glycosaminoglycans. *J. Biol. Chem.* 272 33118–33124. 10.1074/jbc.272.52.33118 9407097

[B13] Huynh M. B. Ouidja M. O. Chantepie S. Carpentier G. Maïza A. Zhang G. (2019). Glycosaminoglycans from Alzheimer’s disease hippocampus have altered capacities to bind and regulate growth factors activities and to bind tau. *PLoS One* 14:e0209573. 10.1371/journal.pone.0209573 30608949 PMC6319808

[B14] Huynh M. B. Rebergue N. Merrick H. Gomez-Henao W. Jospin E. Biard D. S. F. (2022). HS3ST2 expression induces the cell autonomous aggregation of tau. *Sci. Rep*. 12:10850. 10.1038/s41598-022-13486-6 35760982 PMC9237029

[B15] Iqbal K. Alonso Adel C. Chen S. Chohan M. O. El-Akkad E. Gong C. X. (2005). Tau pathology in Alzheimer disease and other tauopathies. *Biochim. Biophys. Acta* 1739 198–210. 10.1016/j.bbadis.2004.09.008 15615638

[B16] Lasagna-Reeves C. A. Castillo-Carranza D. L. Sengupta U. Sarmiento J. Troncoso J. Jackson G. R. (2012). Identification of oligomers at early stages of tau aggregation in Alzheimer’s disease. *FASEB J*. 26 1946–1959. 10.1096/fj.11-199851 22253473 PMC4046102

[B17] Lepinay E. Cicchetti F. (2023). Tau: a biomarker of Huntington’s disease. *Mol. Psychiatry* 28 4070–4083. 10.1038/s41380-023-02230-9 37749233

[B18] Lesné S. Blanchet S. Docagne F. Liot G. Plawinski L. MacKenzie E. T. (2002). Transforming growth factor-beta1-modulated cerebral gene expression. *J. Cereb. Blood Flow Metab*. 22 1114–1123. 10.1097/00004647-200209000-00009 12218417

[B19] Li J.-P. Kusche-Gullberg M. (2016). Heparan Sulfate: biosynthesis, structure, and function. *Int. Rev. Cell Mol. Biol.* 325 215–273. 10.1016/bs.ircmb.2016.02.009 27241222

[B20] Maïza A. Chantepie S. Vera C. Fifre A. Huynh M. B. Stettler O. (2018). The role of heparan sulfates in protein aggregation and their potential impact on neurodegeneration. *FEBS Lett*. 592 3806–3818. 10.1002/1873-3468.13082 29729013

[B21] Maïza A. Sidahmed-Adrar N. Michel P. P. Carpentier G. Habert D. Dalle C. (2020). 3-O-sulfated heparan sulfate interactors target synaptic adhesion molecules from neonatal mouse brain and inhibit neural activity and synaptogenesis in vitro. *Sci. Rep*. 10:19114. 10.1038/s41598-020-76030-4 33154448 PMC7644699

[B22] Minniti J. Checler F. Duplan E. Alves da Costa C. (2025). TFinder: a python web tool for predicting transcription factor binding sites. *J. Mol. Biol*. 437:168921. 10.1016/j.jmb.2024.168921 39842990

[B23] Nakao A. Imamura T. Souchelnytskyi S. Kawabata M. Ishisaki A. Oeda E. (1997). TGF-beta receptor-mediated signalling through Smad2, Smad3 and Smad4. *EMBO J*. 16 5353–5362. 10.1093/emboj/16.17.5353 9311995 PMC1170167

[B24] Nativio R. Lan Y. Donahue G. Sidoli S. Berson A. Srinivasan A. R. (2020). An integrated multi-omics approach identifies epigenetic alterations associated with Alzheimer’s disease. *Nat. Genet*. 52 1024–1035. 10.1038/s41588-020-0696-0 32989324 PMC8098004

[B25] Ouidja M. O. Biard D. S. F. Huynh M. B. Laffray X. Gomez-Henao W. Chantepie S. (2024). Genetic variability in proteoglycan biosynthetic genes reveals new facets of heparan sulfate diversity. *Essays Biochem*. 68 555–578. 10.1042/EBC20240106 39630030 PMC11625870

[B26] Parish C. R. (2006). The role of heparan sulphate in inflammation. *Nat. Rev. Immunol*. 6 633–643. 10.1038/nri1918 16917509

[B27] Ramsden M. Kotilinek L. Forster C. Paulson J. McGowan E. SantaCruz K. (2005). Age-dependent neurofibrillary tangle formation, neuron loss, and memory impairment in a mouse model of human tauopathy (P301L). *J. Neurosci*. 25 10637–10647. 10.1523/JNEUROSCI.3279-05.2005 16291936 PMC6725849

[B28] Rawat P. Sehar U. Bisht J. Selman A. Culberson J. Reddy P. H. (2022). Phosphorylated Tau in Alzheimer’s disease and other tauopathies. *Int. J. Mol. Sci*. 23:12841. 10.3390/ijms232112841 36361631 PMC9654278

[B29] Robbins M. Clayton E. Kaminski Schierle G. S. (2021). Synaptic tau: a pathological or physiological phenomenon? *Acta Neuropathol. Commun.* 9:149. 10.1186/s40478-021-01246-y 34503576 PMC8428049

[B30] Schultheis N. Becker R. Berhanu G. Kapral A. Roseman M. Shah S. (2022). Regulation of autophagy, lipid metabolism, and neurodegenerative pathology by heparan sulfate proteoglycans. *Front. Genet*. 13:1012706. 10.3389/fgene.2022.1012706 36699460 PMC9870329

[B31] Sepulveda-Diaz J. E. Alavi Naini S. M. Huynh M. B. Ouidja M. O. Yanicostas C. Chantepie S. (2015). HS3ST2 expression is critical for the abnormal phosphorylation of tau in Alzheimer’s disease-related tau pathology. *Brain*. 138(Pt 5), 1339–1354. 10.1093/brain/awv056 25842390 PMC5963411

[B32] Siano G. Varisco M. Caiazza M. C. Quercioli V. Mainardi M. Ippolito C. (2019). Tau Modulates VGluT1 Expression. *J. Mol. Biol*. 431 873–884. 10.1016/j.jmb.2019.01.023 30664870

[B33] Snow A. D. Cummings J. A. Lake T. (2021). The unifying hypothesis of Alzheimer’s Disease: heparan sulfate proteoglycans/glycosaminoglycans are key as first hypothesized over 30 years ago. *Front. Aging Neurosci.* 13:710683. 10.3389/fnagi.2021.710683 34671250 PMC8521200

[B34] Song S.-H. Augustine G. J. (2015). Synapsin isoforms and synaptic vesicle trafficking. *Mol. Cells* 38 936–940. 10.14348/molcells.2015.0233 26627875 PMC4673407

[B35] Su J. H. Cummings B. J. Cotman C. W. (1992). Localization of heparan sulfate glycosaminoglycan and proteoglycan core protein in aged brain and Alzheimer’s disease. *Neuroscience* 51 801–813. 10.1016/0306-4522(92)90521-3 1488123

[B36] Thacker B. E. Xu D. Lawrence R. Esko J. D. (2014). Heparan sulfate 3-O-sulfation: a rare modification in search of a function. *Matrix Biol*. 35 60–72. 10.1016/j.matbio.2013.12.001 24361527 PMC4039620

[B37] Tomassoni-Ardori F. Hong Z. Fulgenzi G. Tessarollo L. (2020). Generation of functional mouse hippocampal neurons. *BIO-Protoc* 10 e3702. 10.21769/BioProtoc.3702 32984439 PMC7518151

[B38] Tominaga K. Suzuki H. I. (2019). TGF-β signaling in cellular senescence and aging-related pathology. *Int. J. Mol. Sci.* 20 5002. 10.3390/ijms20205002 31658594 PMC6834140

[B39] Tourville A. Akbar D. Corti O. Prehn J. H. M. Melki R. Hunot S. (2022). Modelling α-synuclein aggregation and neurodegeneration with fibril seeds in primary cultures of mouse dopaminergic neurons. *Cells* 11:1640. 10.3390/cells11101640 35626675 PMC9139621

[B40] Van Horssen J. Wesseling P. Van Den Heuvel L. P. De Waal R. M. Verbeek M. M. (2003). Heparan sulphate proteoglycans in Alzheimer’s disease and amyloid-related disorders. *Lancet Neurol.* 2 482–492. 10.1016/s1474-4422(03)00484-8 12878436

[B41] Wang Y. Mandelkow E. (2016). Tau in physiology and pathology. *Nat. Rev. Neurosci.* 17 22–35. 10.1038/nrn.2015.1 26631930

[B42] Wang Z. Patel V. N. Song X. Xu Y. Kaminski A. M. Doan V. U. (2023). Increased 3- O-sulfated heparan sulfate in Alzheimer’s disease brain is associated with genetic risk gene HS3ST1. *Sci. Adv*. 9:eadf6232. 10.1126/sciadv.adf6232 37235665 PMC10219595

[B43] Wyss-Coray T. Masliah E. Mallory M. McConlogue L. Johnson-Wood K. Lin C. (1997). Amyloidogenic role of cytokine TGF-beta1 in transgenic mice and in Alzheimer’s disease. *Nature* 389 603–606. 10.1038/39321 9335500

[B44] Zhang P. Lu H. Peixoto R. T. Pines M. K. Ge Y. Oku S. (2018). Heparan sulfate organizes neuronal synapses through neurexin partnerships. *Cell* 174 1450–1464.e23. 10.1016/j.cell.2018.07.002. 30100184 PMC6173057

[B45] Zhang Z. Jiang H. Wang Y. Shi M. (2018). Heparan sulfate D-glucosamine 3-O-sulfotransferase 3B1 is a novel regulator of transforming growth factor-beta-mediated epithelial-to-mesenchymal transition and regulated by miR-218 in nonsmall cell lung cancer. *J. Cancer Res. Ther.* 14 24–29. 10.4103/jcrt.JCRT_659_17 29516954

