# Supplementary material for: A novel TGFβ1–Hs3st2–tau axis regulates tau pathology and synaptic integrity
Source: Front Neurosci. 2026 Feb 23;19:1726022. doi: 10.3389/fnins.2025.1726022 (PMC12968242; doi:10.3389/fnins.2025.1726022)
Supplement: Supplementary Figure 1 — Primary hippocampal neurons exhibit aberrant tau phosphorylation at multiple residues and increased Hs3st2 protein expression. (a) Co-Immunofluorescence staining show increased Hs3st2 protein levels (green) and increased abnormal phosphorylation of tau (AT8) in Tg PHN compared to WT. (a.i,a.ii) Quantification of normalized fluorescence intensity confirms a significant increase in Hs3st2 and AT8 immunoreactivity. Error bars represent SEM from 3 independent cultures with 3 fields analyzed by culture. (b) Western blot analysis shows an increased abnormal phosphorylation of tau (MCI, AT8, and PHF1) in Tg PHN compared with WT. (b.i–b.iii) MCI, AT8 and PHF1 quantification of tau abnormal phosphorylation normalized with the loading control GAPDH. Error bars represent SEM from 3 independent cultures analyzed twice. Statistical significance was assessed using Welch’s two-tailed t-test. [file Image_1.pdf]

Supplementary figure 1

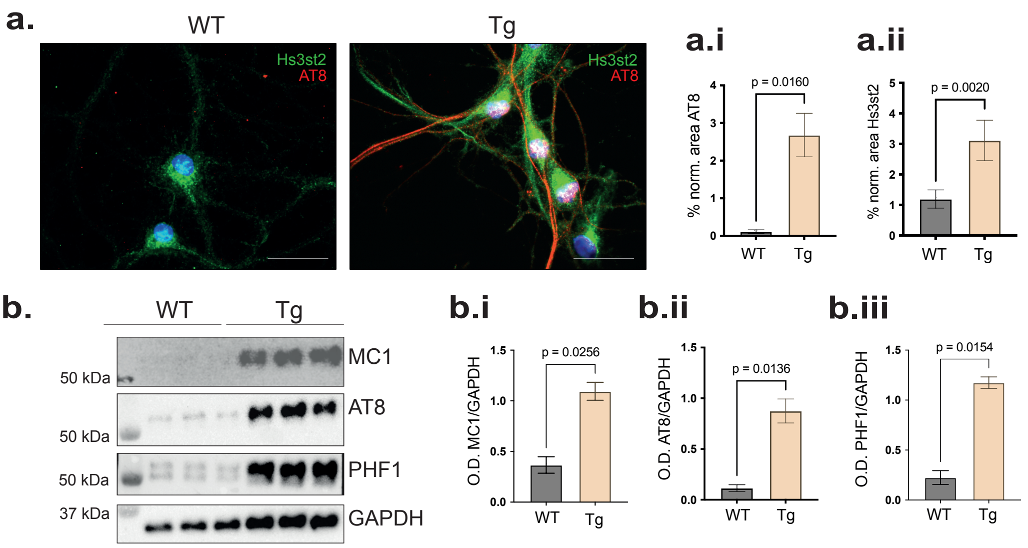

Supplementary figure 1. Primary hippocampal neurons exhibit aberrant tau phosphorylation at multiple residues and increased Hs3st2 protein expression. **a.** Co-Immunofluorescence staining show increased Hs3st2 protein levels (green) and increased abnormal phosphorylation of tau (AT8) in Tg PHN compared to WT. **a.i and a.ii.** Quantification of normalized fluorescence intensity confirms a significant increase in Hs3st2 and AT8 immunoreactivity. Error bars represent SEM from 3 independent cultures with 3 fields analyzed by culture. **b.** Western blot analysis shows an increased abnormal phosphorylation of tau (MCI, AT8 and PHF1) in Tg PHN compared with WT. **b.i, b.ii, and b.iii.** MCI, AT8 and PHF1 quantification of tau abnormal phosphorylation normalized with the loading control GAPDH. Error bars represent SEM from 3 independent cultures analyzed twice. Statistical significance was assessed using Welch's two-tailed t-test.
