# Supplementary material for: A novel TGFβ1–Hs3st2–tau axis regulates tau pathology and synaptic integrity
Source: Front Neurosci. 2026 Feb 23;19:1726022. doi: 10.3389/fnins.2025.1726022 (PMC12968242; doi:10.3389/fnins.2025.1726022)
Supplement: Supplementary Figure 2 — Transcription factor binding site prediction of Smad4 in the promoter region of Hs3st1, Hs3st2, and Hs3st4. (a) Transcription factor binding sites (TFBS) for Smad4 were predicted in the promoter regions of mouse Hs3st1, Hs3st2 and Hs3st4 genes using the TFinder web tool. Position weight matrices (PWMs) for Smad-binding elements (SBEs) were retrieved from the JASPAR database, reconstructed and subsequently employed to scan the promoter regions of Hs3st1, Hs3st2, and Hs3st4 for putative Smad4 transcription factor binding sites. (b) Sequence Retrieval and Analysis Parameters Target genes included three mouse HS sulfotransferase genes: Hs3st1, Hs3st2, and Hs3st4. The relative score threshold was set to 0.85 to ensure high-confidence binding site predictions. (c) Integrative genome viewer and TFBSPred Analysis. Genomic view of chromosome 16 (hg38) showing the Hs3st1, Hs3st2, Hs3st4 gene loci, transcription start sites (TSS), and annotated SBEs. [file Image_2.pdf]

Supplementary figure 2

a.

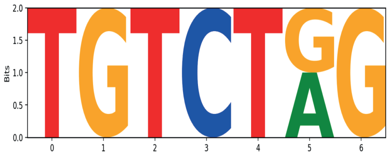

b.

Gene	Position	Score	Rel Score Adj	Score Adjusted	p-value	Region
Hs3st1	3114	8.29	0.90	8.27	4.190000e-04	Promoter
Hs3st2	3825	12.76	1.00	12.36	7.400000e-05	Promoter
Hs3st4	3758	9.93	0.95	10.45	1.380000e-04	Promoter

c.

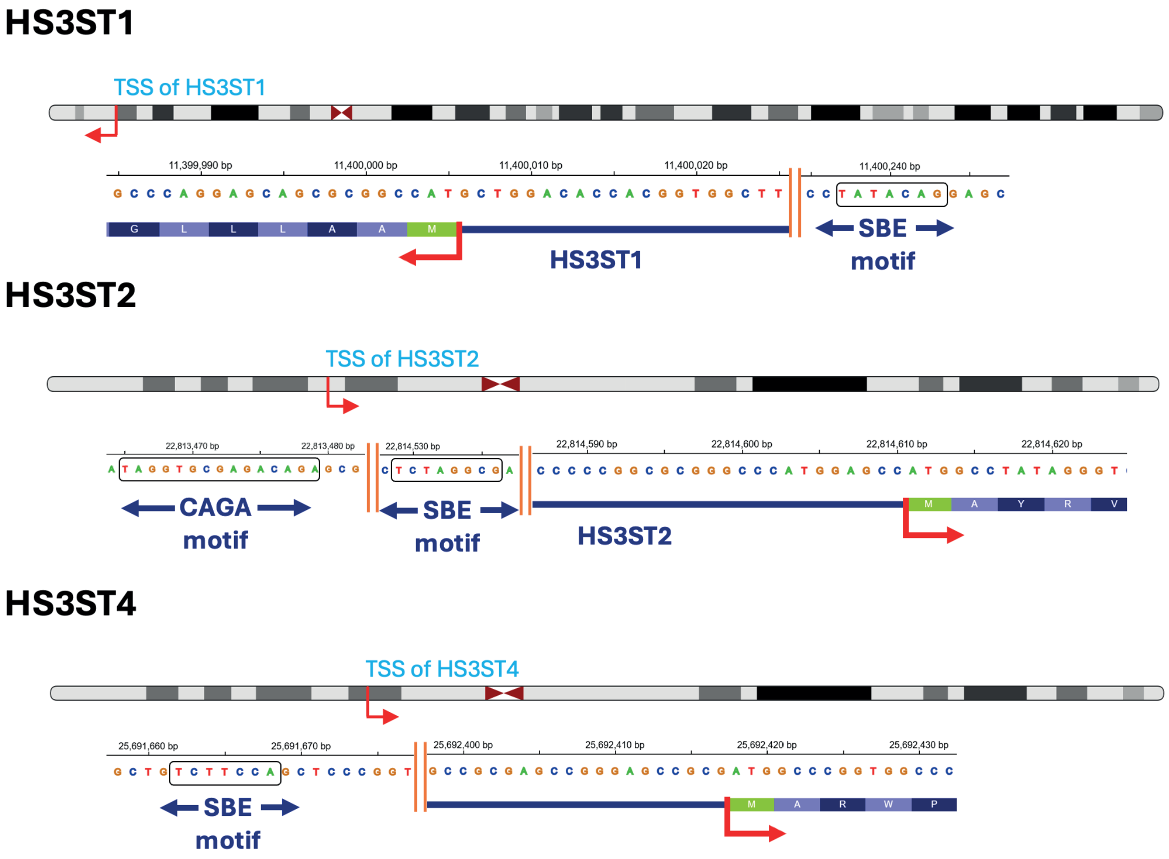

Supplementary figure 2. Transcription factor binding site prediction of Smad4 in the promoter region of Hs3st1, Hs3st2 and Hs3st4. **a.** Transcription factor binding sites (TFBS) for Smad4 were predicted in the promoter regions of mouse Hs3st1, Hs3st2 and Hs3st4 genes using the TFinder web tool. Position weight matrices (PWMs) for Smad-binding elements (SBEs) were retrieved from the JASPAR database, reconstructed and subsequently employed to scan the promoter regions of Hs3st1, Hs3st2, and Hs3st4 for putative Smad4 transcription factor binding sites. **b.** Sequence Retrieval and Analysis Parameters Target genes included three mouse HS sulfotransferase genes: Hs3st1, Hs3st2, and Hs3st4. The relative score threshold was set to 0.85 to ensure high-confidence binding site predictions. **c.** Integrative genome viewer and TFBSPred Analysis. Genomic view of chromosome 16 (hg38) showing the Hs3st1, Hs3st2, Hs3st4 gene loci, transcription start sites (TSS), and annotated SBEs.
