# Supplementary material for: A novel TGFβ1–Hs3st2–tau axis regulates tau pathology and synaptic integrity
Source: Front Neurosci. 2026 Feb 23;19:1726022. doi: 10.3389/fnins.2025.1726022 (PMC12968242; doi:10.3389/fnins.2025.1726022)
Supplement: Supplementary Table 1 — Key resources table. [file Table_1.pdf]

Key Resources Table

Reagent and resources	Sources	Identifier
**Mice**		
CaMK2a-tTa	Jackon Laboratoires (USA)	Stock No. 007004; RRID: MGI:5438794, listed as B6.Cg-Tg (Camk2a-tTA)1Mmay
FVB-*Fgf14*$^{Tg(tetO-MAPT*P301L)4510Kha}$/JlwsJ	Jackson Laboratoires (USA)	Stock No. 015815; RRID: MGI:4819951, listed as Tg(tauP301L)4510 and known as rTg4510)
**Antibodies**		
GAPDH	Thermo Fisher Scientific	AM4300
PHF1	Peter Davis	Gift
MC1	Peter Davis	Gift
T22	Sigma-Aldrich/Merck	ABN454
pSmad2/3	Abcam	ab254407
AT8	Thermo Fisher Scientific	MN1020
Vglut1	Abcam	ab77822
PSD95 (6G6-1C9)	Thermo Fisher Scientific	MA1-045
HS3ST2	Thermo Fisher Scientific	PA5-26522
3S-HS (HS4C3)	Toin van Kuppevelt	Gift (PMID: 16373349)
Synaptophysin	Invitrogen	MA5-14532
**Chemicals and Reagents**		
Neurobasal medium	Gibco	21193-049
B-27 Supplement 50x	Life Technologies	17504044
HBSS	Gibco	14170-088
DNase I	Sigma-Aldrich/Merck	11284932001
Trypsin-EDTA solution	Sigma-Aldrich/ Merck	T3924
HEPES	Sigma-Aldrich/ Merck	H0887
GlutaMAX TM-I	Gibco	35050-038
DMEM + GlutaMAX-I	Gibco	61965-026
Fetal bovine serum	Gibco	10270-098
Penicilin-streptomycin	Gibco	15140-122
Trypsin	Sigma-Aldrich/Merck	T6763
Poly-D-lysine hydrobromide	Sigma-Aldrich/Merck	P7280
DPBS	Thermo Fisher Scientific	14190250
RIPA	Thermo Fisher Scientific	89901
Protease inhibitor cocktail I	Sigma-Aldrich/Merck	P8340
Phosphatase inhibitor cocktail II	Sigma-Aldrich/Merck	P5726
Phosphatase inhibitor cocktail III	Sigma-Aldrich/Merck	P0044
Beta-Mercaptoethanol	Sigma-Aldrich/Merck	M6250
4x Laemmli SDS	GeneTex	GTX16355
Tween-20	Sigma-Aldrich/Merck	P9416
Triton-X100	VWR	28 817.295
3,3′-Diaminobenzidine tetrahydrochloride hydrate (DAB)	Sigma-Aldrich/Merck	D5637
Bovine serum albumin (BSA)	Vector laboratories	SP-5050-500
DAPI	Thermo Fischer Scientific	D1306
ProLong^{TM} Gold antifade mountant	Thermo Fischer Scientific	P10144

Nuclease-free water	Thermo Fisher Scientific	AM9937
TRIzol® reagent	Thermo Fisher Scientific	15596018
TGFβ receptor inhibitor (SB431541)	Sigma-Aldrich/Merck	S4317-5MG
TGFβ1	Thermo Fisher Scientific	100-21C
**Critical Commercial Assays and Kits**		
AffinityScript cDNA synthesis kit	Agilent Technologies	200436
Brilliant III ultra-fast SYBR®	Agilent Technologies	600882
NucleoSpin RNA XS	Macherey-Nagel	740902.50
RNeasy Minikit	QIAGEN	74104
Elite ABC KIT	Vector Laboratories	PK-6100
PIERCE™ BCA Protein Assay Kit	Thermo Scientific	23225
Clarity Western ECL Substrate	Bio-Rad	1705060
**Recombinant DNA**		
MISSION® Lentivirus Transduction Particles	Sigma-Aldrich/Merck	TRCN0000241592
MISSION® Lentivirus Transduction Particles (Non-Target Control)	Sigma-Aldrich/Merck	SHC016V
